# Measurement invariance of the inventory of Callous‑Unemotional traits in different age groups from preschool age to late adolescence in Germany

**DOI:** 10.1186/s40359-024-01789-4

**Published:** 2024-05-27

**Authors:** Annika Rademacher, Neele Bäker, Jule Eilts, Ute von Düring, Jessica Wilke

**Affiliations:** 1 Department of Special Needs Education and Rehabilitation, Carl von Ossietzky Universität Oldenburg, Ammerlaender Heerstraße 114-118, 26129 Oldenburg, Germany; 2 Ludwig-Maximilians Universität München Developmental Psychology, Leopoldstr. 13, 80802 Munich, Germany

**Keywords:** Callous-unemotional traits, Psychopathy, Measurement invariance

## Abstract

**Background:**

Callous-unemotional traits are associated with the development of severe behavior problems, delinquency, and psychopathy. Previous studies have repeatedly shown that CU traits may be present as early as preschool age, and they have consistently used the Inventory of Callous-Unemotional Traits (ICU) to assess CU traits in children and adolescents. A three-factor structure for the ICU has been widely endorsed.

**Method:**

The aim of our study is to compare the three-factor structure of the ICU in different age groups (preschool, middle childhood, early, and late adolescence) and to test for measurement invariance in a German sample of *N* = 2368 children and adolescents (*M* = 11.76 years; *SD* = 3.72).

**Results:**

The results of our study indicate configural measurement invariance, suggesting that the ICU has the same structure in all age groups but with different meanings, parameters, and mean values in the groups.

**Conclusion:**

Accordingly, the ICU cannot be applied in the same way to children and adolescents of different age groups, which emphasizes the need for a more differentiated assessment.

**Supplementary Information:**

The online version contains supplementary material available at 10.1186/s40359-024-01789-4.

## Background

CU traits (Callous-unemotional traits) are defined as affective-social deficits that characterize an extreme form of aggressive-dissocial behavior, marked by a pronounced inclination towards violence [1]. CU traits are predominantly examined in the context of psychopathy and are part of the concept of psychopathy in adulthood, but can occur as early as preschool age [2, 3]. Children and adolescents with callous-unemotional traits are characterized by a deficit in empathy and guilt, a tendency to manipulate others for personal benefit, and the expression of superficial emotions [4]. Specifically, CU traits identify a subgroup of children and adolescents characterized by persistent and severe conduct problems [4, 5]. These traits play a crucial role in capturing the affective dimension of psychopathy, functioning alongside other dimensions of psychopathic traits [1, 6]. Research has highlighted the significance of CU traits in relation to developmental outcomes like conduct problems [7, 8], impulsivity [9], aggressive behavior, and delinquency [10, 11].

### The Inventory of Callous‑Unemotional traits (ICU)

To assess callous-unemotional traits, the Inventory of Callous-Unemotional Traits (ICU) [12] is commonly used in research studies [e.g., 13–16]. The questionnaire was designed to measure the callous and unemotional components of psychopathy. According to the ICU [12], callous-unemotional traits can be defined as a combination of three dimensions: (1) callousness, which involves a lack of concern or remorse for others; (2) uncaring, which refers to a lack of interest in others or one’s own performance; and (3) unemotionality, which involves a difficulty in expressing emotions [15]. Research conducted in various age groups and countries has also revealed alternative factor structures [e.g., 13, 17, 18], but the three-factor model has been consistently confirmed for different age groups (preschool: Ezpeleta et al. [2]; elementary school: Waller et al. [19]; adolescents: Pihet et al. [20], Roose et al. [21]), and methodologically operationalized to provide the best model fit in various samples [15, 22]. The meta-analysis by Cardinale and Marsh [22] shows satisfactory pooled Cronbach’s alpha values for the three subscales (callousness$: \stackrel{-}{\alpha }$ = 0.75, uncaring: $\stackrel{-}{\alpha }$ = 0.80, unemotional: $\stackrel{-}{\alpha }$ = 0.71) and the total ICU ($\stackrel{-}{\alpha }$ = 0.83). Additionally, their analysis points to the high validity of the ICU [22]. The three-factor model emerges as the model with the best fit for self-reported CU traits [15, 22] as well as parent- or caregiver-reported CU traits [2, 19].

The ICU is the only known measure that focuses solely on CU traits in children and adolescents including a three-factor structure. The ICU comes in five versions: ICU-Parent, ICU-Teacher, ICU-Youth, ICU-Parent Preschool and ICU-Teacher Preschool. However, despite these variations it does consist of the same 24 items (some items were formulated slightly differently through the versions while maintaining the same meaning) in each version and is divided into the three subscales measuring callousness, uncaring, and unemotional traits [12].

### Callous-unemotional traits in different age groups

Studies highlighted that callous-unemotional traits are heritable, with estimates ranging from 36 to 67% [23]. Candidate gene studies link CU traits to the serotonin and oxytocin systems, while epigenetic changes in these genes are also associated with CU traits (see Moore et al. [23] for review). CU traits develop in early childhood (see Frick et al. [14] for review) and are moderately stable [4, 24]. In studies, aside from biological factors, environmental factors like parenting and attachment were also identified as crucial contributors to the development of callous-unemotional traits [25–27]. These environmental factors may be linked with the rise and stability of callous-unemotional traits [25].

During preschool age, children are still developing social-emotional competencies and learning how to regulate their emotions. Kimonis et al. [28] were able to show that children under six years of age who score high on the ICU show poor recognition of facial expressions, are less attentive towards distress cues, and are more likely to be antisocial, aggressive, and high on other psychopathy dimensions. However, preschoolers may also show some behaviors that resemble callous-unemotional traits, like a lack of remorse or guilt. These behaviors are typically not indicative of a stable personality trait and may be related to age-appropriate cognitive and emotional development, it’s important to recognize that personality can evolve over time as a function of development [18]. Nevertheless, Longman et al. [8] identified a large effect size for the relationship between conduct problem severity and callous-unemotional traits in early childhood.

In middle childhood and by elementary school age, children are better able to regulate their emotions and display more stable antisocial behavior. Even though antisocial behavior is less common in middle childhood than in adolescents [29, 30], some children may still exhibit callous-unemotional traits. These children may be at greater risk of developing conduct disorder [14].

During adolescence, the risk to engage in risky behavior and exhibit delinquent behavior increases [4, 31, 32]. Adolescents with callous-unemotional traits may display an increased tolerance for risk and may be less responsive to punishment [33]. Pihet et al. [20] discuss that ICU validation studies yielded different results regarding analyses of age differences in early and late adolescents: In the study by Essau et al. [15], 15- to 16-year-olds showed higher ICU scores compared to 13- to 14-year-olds and 17- to 18-year-olds. Ciucci et al. [13] identified higher ICU scores for eighth graders compared to sixth graders (with an overall age range from 10 to 16 years), while White et al. [34] did not find age differences for detained male adolescents, and Pihet et al. [20] did not find age differences in a community and institutionalized (youth welfare or juvenile justice institutions) sample of adolescents.

### Current study

Our study aims to investigate the measurement invariance of the Inventory of Callous-Unemotional Traits (ICU) across preschool to late adolescence. By assessing measurement invariance, we aim to determine if the ICU can reliably measure CU traits across these age groups. Group validity analysis will assess whether the ICU consistently measures as intended across different age groups, ensuring fair assessments [35]. We’ll specifically examine the ICU’s three-factor structure, as previous research and the author [12] suggests its suitability. Analyzing this structure across different age groups will help determine its validity and reliability throughout childhood and adolescence, anticipating successful replication with a German sample.

## Method

### Participants and procedure

The data collection stems from larger projects led by the authors with the central purpose of systematically analyzing emotional, social, and behavioral development in children and adolescence. The ICU was assessed as part of a larger battery of tests. We report only those instruments and data relevant to the current research questions. The data presented are quantitative cross-sectional data.

Informed consent was obtained from the relevant daycare center management, the school board, and the principals of the participating schools. To recruit the sample, northern German schools and daycare centers were contacted or called and informed about the study. If the schools and daycare centers were interested in participating, information flyers and consent forms were distributed to the participating children and adolescents, guardians, and preschool teachers. In addition, a positive vote from the relevant Institutional Review Board was available. The data were collected between 2016 and 2021. All participants and their guardians were informed about the study, the voluntary nature of participation, and the confidentiality of their data and gave their active written consent to participate. Participants (children and teachers) were told that they can withdraw from the study at any time or skip any questions that they do not want to answer. No incentives were offered for participation.

A total of *N* = 2368 children and adolescents (51.5% male) with an average age of *M* = 11.76 years (*SD* = 3.72, *Min* = 5, *Max* = 19) took part in the study. Table 1 shows the demographic variables by age group.

**Table 1:** Demographic variables

	Preschool age	Middle childhood	Early adolescence	Late adolescence
*N*	498	631	646	593
Sex male (%)	256 (51.41)	336 (53.25)	323 (50.00)	299 (50.42)
Mean age (*SD*)	5.69 (0.62)	10.89 (1.22)	13.56 (0.50)	15.83 (1.09)
Age range in years	5–7	8–12	13–14	15–19

*Note* SD = Standard deviation

### Instruments

To measure callous-unemotional traits in childhood and adolescence, we utilized the German version of the Inventory of Callous-Unemotional Traits (ICU; [12]; German version by Essau et al. [15]). The ICU consists of 24 items (e.g., not caring to hurt someone; 11 items), uncaring (e.g., trying to do the best (reverse scoring); 8 items), and unemotionality (e.g., not showing emotions; 5 items) and previous research in both international and German samples have shown a three-factor structure (callousness, uncaring, unemotional) [e.g., 4, 15, 22, 36]. The items of the ICU were rated on a four-point scale ranging from (1) not at all true to (4) definitely true, with items requiring reverse scoring being recoded. Thus, higher scores on each dimension indicate higher levels of callousness, uncaring, and unemotionality. It is important to note that we assessed callous-unemotional traits through different sources of assessment, including preschool teacher-report and self-report measures. Therefore, slightly different versions of the ICU were used, and some items were formulated slightly differently while maintaining the same meaning. For preschool children in group one (*N* = 498), an external report from preschool teachers was chosen to assess CU traits, while for children and adolescents in the other three age groups (middle childhood, early and late adolescence), a self-report was used. Studies on callous-unemotional traits in middle childhood incorporate self-reports [e.g., 13] and other-reports [e.g., 30].We assume that we will arrive at more valid answers if we let the children answer the items themselves at an older age.

### Data analytic procedure

#### Confirmatory factor analysis

In the statistical analyses, the factor structure of the ICU is first examined for each age group. The subsamples comprise *N* = 498 for preschool aged children, *N* = 631 for middle childhood, *N* = 646 for early adolescence, and *N* = 593 for late adolescence. Confirmatory factor analyses are therefore performed for each of the four groups individually (preschool age, middle childhood, early adolescence, and adolescence). For the confirmatory testing, the model structure of the ICU is examined as specified by Frick [12]. A three-factor model with the correlated factors *callousness, uncaring* and *unemotional* is assumed. Due to its statistical weakness (low factor loading), item 10, “I do not let my feelings control me” is excluded from the analyses, as it has also been handled in previous research [e.g., 2]. Error correlations are allowed for items with similar content statements (15*23, 4*17, 8r*17, 11*20, 3*23, 16*17, 1*19, 8*24, 12*6, 12*22, 6*19, 11*3, 8*21, 6*22, 21*17, 5*18, 8*5, 4*16, 8*16, 12*14, 2*9, 2*8, 2*23, 18*20, 2*24, 8r*1, 17*1, 21*16, 3*15, 13*14, 9*24, 23*24, 11*24, 4*8, 13*16, 19*22, 20*23, 20*15, 3*20, 9*6, 11*12, 9*15, 1*12, 9*5, 12*19, 17*24, 8*9, 3*16, 5*22, 9*21, 6*7, 8*19, 8*15, 7*24, 1*22). The *Root Mean Square Error of Approximation* (RMSEA), the *Comparative Fit Index* (CFI), and the *Tucker-Lewis Index* (TLI) are used to evaluate the model quality. *RMSEA* values < 0.08 and *CFI* and *TLI* values > 0.90 represent a good model fit [37]. In addition, *Chi*^*2*^ values are reported; however, the sensitivity of *Chi*^*2*^ for larger samples (*N* > 200) needs to be considered [38]. The *Standardized Root Mean Squared Residual* (SRMR) is not calculated due to missing values. Data with completely missing values are excluded from the analyses in advance (*N* = 124). Individual missing values are estimated within the model estimation using *Full Information Maximum Likelihood* (FIML; [39, 40]). Individual missing data exists for less than 9% of the sample. *FIML* provides reliable estimates even if the data is not normally distributed [41] or when estimating ordinal data [42]. To reduce the probability of alpha-errors, *p*-values are FDR-corrected (false discovery rate; [43]), which represents a liberal method in multiple testing in structural equation modeling [44]. The reliability was assessed using Cronbach’s alpha for the best fitting model for the different age groups.

#### Measurement invariance

Confirmatory multi-group factor analyses (MGCFA) are used to test for measurement invariance (MI) in the ICU for all four age groups. The sample size of the four groups for measurement invariance testing is between *N* = 498 for preschool children and *N* = 646 for early adolescents which exceeds the minimum sample size of *N* = 200 per group and is therefore sufficiently large [45]. According to Koh and Zumbo [45], different group sizes up to a ratio of 200:800 are not an obstacle for the test of measurement invariance. If the measurement model shows an acceptable fit in all groups, MI is tested at three different levels successively: configural, metric, and scalar [46]. The levels of MI are distinguished by varying model restrictions, which depend on whether factor loadings, intercepts, and residual variance are equivalent across groups [47]. To achieve configural measurement (first level of MI) invariance, only the factor structure in all groups must be equivalent, which means that the construct of CU traits being assessed by using the ICU-Questionnaire has a similar structure across all four groups The next level of metric measurement invariance is characterized by additional identical factor loadings across groups, implying that that each observed indicator (item) refers with similar strength to the latent construct being assessed. To achieve scalar invariance (the third level of MI), the factor structure has a similar structure across groups, and factor loadings and intercepts are constrained to be equal. Achieving scalar MI indicates that an identical observed score refer to a similar true score across groups [47]. In order to subsequently interpret group means, the residual variances must also be identical [47]. To decide whether measurement invariance is present for the ICU in each age group, changes in Root Mean Squared Error of Approximation (∆RMSEA) along with changes in Comparative Fit Index (∆CFI) and Tucker-Lewis Index (∆TLI) are observed. According to Chen [48], a model represents the data structure equally well in the different groups if the CFI does not decrease by more than 0.01 and the RMSEA does not increase by more than 0.015 from the configural to the metric model and from the metric model to the scalar model, respectively (and from the scalar to residual invariance). *Full Information Maximum Likelihood* was used for model estimation [39, 40]. All analyses are conducted using STATA 18.

## Results

Prior to confirmatory testing of the ICU structure and measurement invariance, descriptive statistics and intercorrelations are calculated and presented in Table 2 and Table S 1. Item intercorrelations of each proposed factor are significant (with one exception for item 2 and item 8).

**Table 2:** Descriptive Statistics of the ICU items for each age group

Item	Preschool age			Middle childhood			Early adolescence			Late adolescence		
	*M*	*SD*	Range	*M*	*SD*	Range	*M*	*SD*	Range	*M*	*SD*	Range
1. I express my feelings openly^r^.	2.775	0.858	1–4	2.478	0.908	1–4	2.236	0.844	1–4	2.335	0.892	1–4
2. What I think is “right” and “wrong” is different from what other people think.	1.276	0.638	1–4	2.009	0.965	1–4	2.285	0.883	1–4	2.256	0.830	1–4
3. I care about how well I do at school or work^r^.	3.126	0.794	1–4	3.267	0.854	1–4	3.151	0.851	1–4	3.233	0.814	1–4
4. I do not care who I hurt to get what I want.	1.463	0.805	1–4	1.255	0.660	1–4	1.371	0.718	1–4	1.344	0.644	1–4
5. I feel bad or guilty when I do something wrong^r^.	2.644	0.894	1–4	3.079	0.955	1–4	3.061	0.944	1–4	3.080	0.938	1–4
6. I do not show my emotions to others.	1.340	0.650	1–4	2.150	0.861	1–4	2.334	0.956	1–4	2.316	0.931	1–4
7. I do not care about being on time.	1.597	0.915	1–4	1.457	0.894	1–4	1.547	0.856	1–4	1.480	0.813	1–4
8. I am concerned about the feelings of others^r^.	2.871	0.854	1–4	3.231	0.894	1–4	3.198	0.824	1–4	3.247	0.804	1–4
9. I do not care if I get into trouble.	1.369	0.723	1–4	1.756	0.996	1–4	1.806	0.957	1–4	1.873	0.955	1–4
10. I do not let my feelings control me.	1.880	0.857	1–4	2.276	0.989	1–4	2.271	0.905	1–4	2.328	0.890	1–4
11. I do not care about doing things well.	1.607	0.956	1–4	1.595	0.926	1–4	1.634	0.829	1–4	1.544	0.789	1–4
12. I seem very cold and uncaring to others.	1.162	0.468	1–4	1.517	0.806	1–4	1.754	0.901	1–4	1.909	0.938	1–4
13. I easily admit to being wrong^r^.	2.357	0.917	1–4	2.471	1.000	1–4	2.294	0.893	1–4	2.382	0.906	1–4
14. It is easy for others to tell how I am feeling^r^.	2.921	0.872	1–4	2.309	0.954	1–4	2.094	0.850	1–4	2.052	0.832	1–4
15. I always try my best^r^.	3.099	0.813	1–4	3.461	0.759	1–4	3.245	0.823	1–4	3.189	0.823	1–4
16. I apologize (“say I am sorry”) to persons I hurt^r^.	3.269	0.807	1–4	3.512	0.781	1–4	3.313	0.852	1–4	3.346	0.798	1–4
17. I try not to hurt others’ feelings^r^.	2.958	0.882	1–4	3.408	0.845	1–4	3.337	0.806	1–4	3.355	0.790	1–4
18. I do not feel remorseful when I do something wrong.	1.503	0.790	1–4	1.658	0.941	1–4	1.749	0.953	1–4	1.711	0.898	1–4
19. I am very expressive and emotional^r^.	2.698	0.989	1–4	2.655	1.019	1–4	2.393	0.975	1–4	2.606	0.951	1–4
20. I do not like to put the time into doing things well.	1.777	0.924	1–4	1.904	0.984	1–4	1.920	0.858	1–4	1.798	0.839	1–4
21. The feelings of others are unimportant to me.	1.547	0.818	1–4	1.308	0.687	1–4	1.345	0.680	1–4	1.376	0.706	1–4
22. I hide my feelings from others.	1.799	0.867	1–4	2.198	1.024	1–4	2.386	0.995	1–4	2.327	0.925	1–4
23. I work hard on everything I do^r^.	3.061	0.845	1–4	2.898	0.961	1–4	2.747	0.872	1–4	2.735	0.859	1–4
24. I do things to make others feel good^r^.	2.369	0.871	1–4	2.988	0.908	1–4	2.820	0.863	1–4	2.843	0.881	1–4

*Note* M = Mean; *SD* = Standard deviation; ^*r*^ = reverse-coded items

### Confirmatory factor analysis

**Fig. 1:**
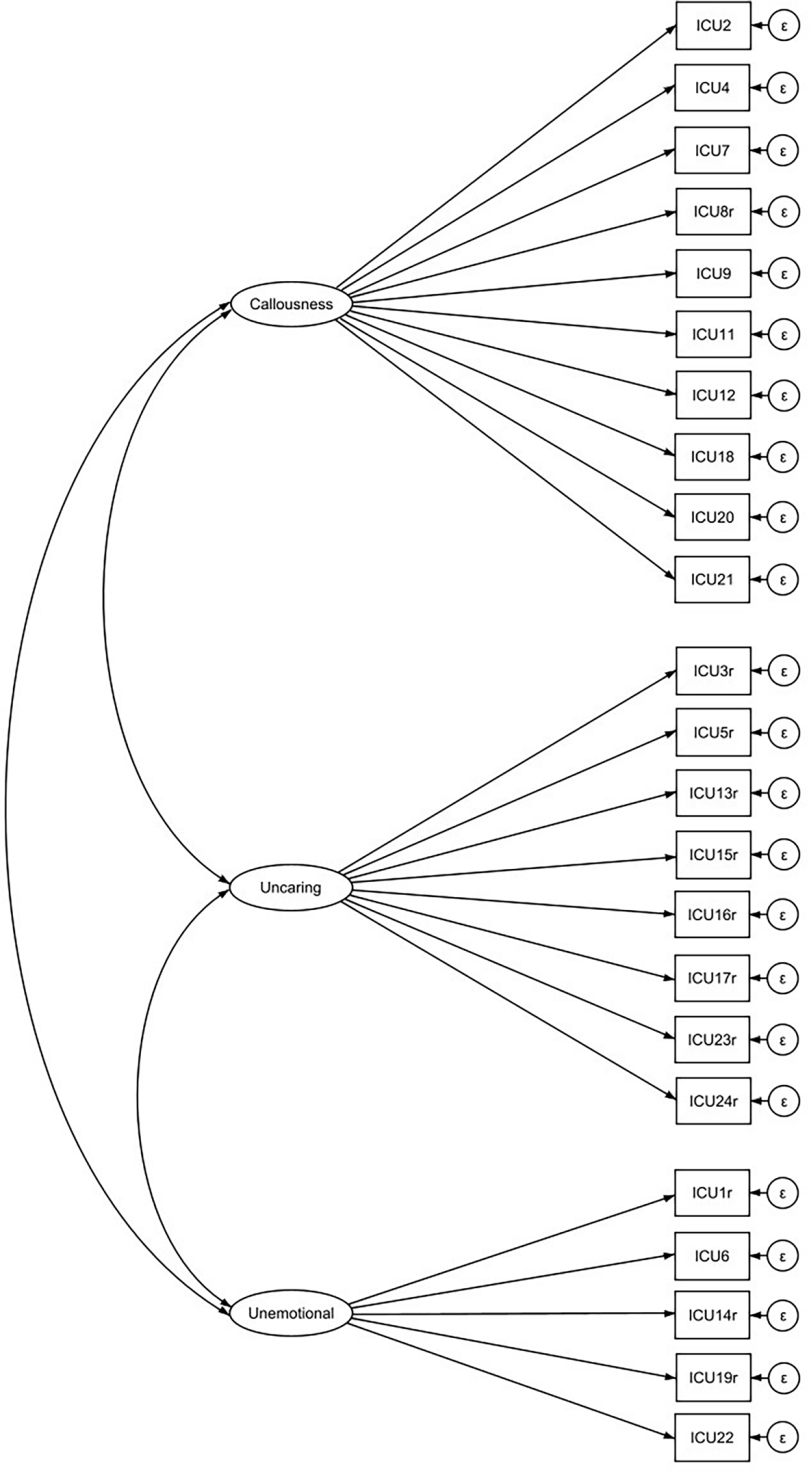
Measurement model of the ICU. *Note* Item labels in Table 2; *r* = reverse-coded items; error correlations are not displayed

For confirmatory testing of the ICU model structure, a three-factor model with the latent factors callousness, uncaring, and unemotional is tested. The goal is to find a model that represented the ICU structure equally well in all four groups (preschool age, middle childhood, early adolescence and late adolescence). Figure 1 illustrates the measurement model, which represents a good model fit across groups (Table 3). Table 4 summarizes the factor loadings and intercepts for each age group. All indicator items load significantly on each factor (with one exception for item 22 in middle childhood). The reliability was assessed using Cronbach’s alpha for the different age groups. Adequate values were found and presented in Table 3.

**Table 3:** Model fit indices of the ICU measurement model for each age group

						Cronbach‘s Alpha		
**Group**	***χ*** ^***2***^	***df***	***RMSEA***	***CFI***	***TLI***	**Callousness**	**Uncaring**	**Unemotional**
Preschool age	404.031***	172	0.054	0.938	0.909	0.76	0.84	0.78
Middle childhood	344.347***	172	0.042	0.928	0.900	0.72	0.72	0.62
Early adolescence	380.655***	172	0.044	0.935	0.904	0.72	0.74	0.72
Late adolescence	375.176***	172	0.045	0.934	0.902	0.73	0.74	0.72

*Note χ*^*2*^ = Chi-Square statistic; *df* = degrees of freedom; *CFI* = Comparative Fit Index; *RMSEA* = Root Mean Square Error of Approximation; *TLI* = Tucker-Lewis Index; *p < .05; **p < .01; ***p < .001

**Table 4:** Factor loadings and intercepts of the measurement model of the ICU for each age group

	Preschool age				Middle childhood				Early adolescence				Late adolescence			
Item	IC	FL	SD	95% CI	IC	FL	SD	95% CI	IC	FL	SD	95% CI	IC	FL	SD	95% CI
1	2.613	0.650***	0.044	0.564-0.735	2.828	0.794***	0.128	0.543-1.045	3.334	0.835***	0.055	0.728-0.942	3.001	0.672***	0.067	0.541-0.803
2	2.001	0.406***	0.045	0.313-0.490	2.083	0.225***	0.047	0.132-0.317	2.587	0.288***	0.045	0.200-0.376	2.719	0.307***	0.045	0.218-0.395
3	2.365	0.539***	0.039	0.462-0.616	2.019	0.373***	0.046	0.283-0.462	2.183	0.344***	0.043	0.259-0.429	2.171	0.383***	0.045	0.295-0.471
4	1.819	0.632***	0.034	0.566-0.698	1.910	0.621***	0.034	0.555-0.688	1.912	0.519***	0.037	0.446-0.592	2.092	0.483***	0.039	0.406-0.559
5	2.644	0.463***	0.042	0.381-0.546	1.897	0.476***	0.040	0.398-0.555	2.061	0.526***	0.036	0.456-0.597	2.052	0.539***	0.038	0.465-0.613
6	2.080	0.617***	0.044	0.532-0.703	2.251	0.443***	0.074	0.299-0.587	2.447	0.575***	0.046	0.485-0.666	2.490	0.727***	0.072	0.568-0.867
7	1.747	0.394***	0.045	0.306-0.481	1.709	0.475***	0.040	0.398-0.553	1.812	0.441***	0.040	0.362-0.520	1.824	0.403***	0.041	0.322-0.484
8	2.474	0.686***	0.034	0.619-0.752	1.984	0.319***	0.052	0.217-0.420	2.216	0.420***	0.047	0.328-0.512	2.207	0.449***	0.044	0.363-0.536
9	1.891	0.505***	0.042	0.422-0.588	1.777	0.353***	0.040	0.457-0.613	1.890	0.466***	0.042	0.383-0.549	1.963	0.490***	0.040	0.411-0.570
11	1.682	0.219***	0.049	0.122-0.315	1.725	0.515***	0.040	0.437-0.593	1.974	0.474***	0.040	0.395-0.554	1.962	0.570***	0.038	0.496-0.645
12	2.487	0.500***	0.040	0.421-0.579	1.907	0.550***	0.038	0.475-0.624	1.968	0.486***	0.039	0.409-0.563	2.037	0.534***	0.039	0.458-0.610
13	2.898	0.591***	0.037	0.517-0.664	2.553	0.233***	0.047	0.141-0.325	3.033	0.156***	0.045	0.069-0.244	2.894	0.150**	0.047	0.058-0.242
14	2.382	0.650***	0.043	0.566-0.733	2.827	0.227**	0.071	0.089-0.365	3.441	0.362***	0.047	0.270-0.453	3.544	0.344***	0.047	0.251-0.437
15	2.355	0.598***	0.035	0.528-0.667	2.041	0.595***	0.037	0.523-0.667	2.140	0.567***	0.036	0.498-0.637	2.203	0.434***	0.042	0.352-0.516
16	2.152	0.761***	0.031	0.702-0.821	1.930	0.673***	0.037	0.601-0.745	1.989	0.678***	0.034	0.612-0.744	2.094	0.616***	0.039	0.538-0.693
17	2.336	0.718***	0.033	0.653-0.782	1.908	0.598***	0.043	0.514-0.681	2.083	0.657***	0.037	0.584-0.730	2.096	0.719***	0.039	0.642-0.796
18	1.907	0.673***	0.032	0.611-0.735	1.774	0.429***	0.041	0.348-0.509	1.840	0.373***	0.041	0.292-0.454	1.905	0.480***	0.039	0.404-0.555
19	2.337	0.544***	0.056	0.434-0.653	2.335	0.801***	0.178	0.454 − 1.150	2.700	0.660***	0.084	0.495-0.826	2.530	0.725***	0.129	0.472-0.977
20	1.939	0.342***	0.046	0.251-0.432	1.977	0.429***	0.0418	0.348-0.511	2.240	0.380***	0.042	0.297-0.463	2.148	0.312***	0.046	0.221-0.403
21	1.894	0.703***	0.031	0.641-0.765	1.909	0.494***	0.040	0.416-0.572	1.955	0.615***	0.036	0.544-0.686	1.953	0.550***	0.038	0.476-0.625
22	2.076	0.635***	0.052	0.533-0.737	2.156	0.090	0.130	− 0.165-0.343	2.410	0.408***	0.074	0.263-0.552	2.514	0.647***	0.101	0.449-0.844
23	2.297	0.564***	0.037	0.490-0.637	2.185	0.309***	0.047	0.217-0.401	2.588	0.347***	0.042	0.264-0.429	2.631	0.282***	0.045	0.194-0.371
24	3.042	0.580***	0.038	0.505-0.654	2.238	0.470***	0.042	388-0.553	2.558	0.482***	0.040	0.404-0.560	2.474	0.524***	0.043	0.440-0.607

*Note* Item labels in Table 3; *IC* = Intercept; *FL* = Factor loading (standardized); *SD* = Standard deviation; *CI* = Confidence interval; *p < .05; **p < .01; ***p < .001; *FDR*-corrected *p*-values

### Measurement invariance

The model fit indices allow the assumption of configural measurement invariance of the ICU across the age groups (*RMSEA* < 0.08; *CFI* and *TLI* > 0.90). In comparing the configural and the metric model, the RMSEA suggests metric invariance (∆*RMSEA* ≤ 0.015), while the *CFI* and *TLI* indicate only configural invariance (∆*CFI* ≥ 0.010; ∆*TLI* ≥ 0.010). In the next step, scalar invariance is therefore analyzed. The model fit comparison shows clearly worse *RMSEA* (∆*RMSEA* ≥ 0.015) as well as worse *CFI* and *TLI* values (∆*CFI* ≥ 0.010; ∆*TLI* ≥ 0.010), so that the results do not confirm the presence of a scalar measurement invariance. When comparing the differences in the fit indices of the configural and metric models, the configural model is chosen after a conservative decision [48]. Complete measurement invariance of the ICU across the four different age groups could not be achieved, results only indicate configural measurement invariance. The values of the measurement invariance testing are presented in Table 5.

**Table 5:** Measurement invariance of the ICU across age groups

Model	χ^2^	df	RMSEA	CFI	TLI	∆RMSEA	∆CFI	∆TLI
Configural	1504.210***	688	0.046	0.934	0.903			
Metric	1838.202***	748	0.051	0.912	0.881	+ 0.005	− 0.022	− 0.022
Scalar	3227.806***	808	0.073	0.805	0.756	+ 0.022	− 0.107	− 0.125

*Note χ*^*2*^ = Chi-Square statistic; *df* = degrees of freedom; *CFI* = Comparative Fit Index; *RMSEA* = Root Mean Square Error of Approximation; *TLI* = Tucker-Lewis Index; *p < .05; **p < .01; ***p < .001

## Discussion

Our study’s aim was to assess whether the ICU [12] measures CU traits consistently across different age groups from preschool to late adolescence. The results indicate configural measurement invariance. Accordingly, the construct of CU traits measured with the ICU has a similar structure across age groups. However, the latent and manifest variables have different meanings in the groups, and the parameters and mean values differ [47]. The results suggest that individual items are understood differently by different age groups. In other words, items are interpreted differently by preschool teachers, middle-aged children, and young and older adolescents. The results indicate that the ICU cannot be interpreted uniformly for children and adolescents of different age groups. Especially when looking at the different intercepts of the items of the unemotional factor, it becomes clear that there are large differences between the age groups here (e.g., “not showing emotions; item 6”). But, also, the intercepts for items of the callousness and uncaring factors differ strongly among the age groups for some items. For example, there are differences in the understanding of the item “feeling bad or guilty when doing something wrong; item 5” of the uncaring factor. Even though studies highlighted moderate stability for CU traits [4, 24], children may differ in the developmental precursor skills that are associated with CU traits. It is possible that younger children lack competencies on a cognitive or social-emotional level. Item 5 for example describes empathy or the ability to take the perspective of others, which may not be fully developed in younger children. Factor analytic studies for children (e.g., in middle childhood, Hawes et al. [5] and in preschoolers, Zumbach et al. [18]), found a best fitting factor structure that excluded most unemotional items. Similar, Kimonis et al. [49] discuss that the unemotional item set may need refining for young children. The situation is similar, for example, for the callousness factor item “concerned about the feelings of others; item 8”. Different meanings of items may be due to different stages of the development of children in different age groups, from preschool age to late adolescence. Possibly, the items may reflect developmental phenomena in younger children [cf. 18], transitioning to expressions of CU traits in older adolescence.

### Implications

As just indicated, according to our findings, the ICU cannot be interpreted identically to children and adolescents of different age groups. This highlights the need for a more differentiated assessment. Frick et al. [14] already provided indications that the instrument for capturing CU traits is not yet fully exhaustive. Our results show that the ICU exhibits similarly good reliabilities across age groups and that the factor structure can also be replicated. However, we need to better understand CU traits, especially at young ages, by following and considering developmental trajectories from early preschool age to adolescence. So far, the use of the same instrument for all samples, from early childhood to adolescence, has assumed that all children and adolescents have the same developmental prerequisites for understanding and answering the items presented.

Many items in the ICU refer to competencies that are closely linked to social-emotional and partly cognitive development. However, these competencies are far from complete in childhood, so difficulties can arise in differentiating between CU traits and social-emotional developmental deficits. For example, items focusing on remorse, such as apologizing when hurting others, or attempting to make amends, are closely linked to feelings of shame and guilt. Shame and guilt are described as intrapersonal emotions and are considered complex emotions that are formed in childhood [50, 51]. Children are even not able to verbally distinguish between guilt and shame until they are about 10–11 years old [52]. Younger children may still have difficulties experiencing these emotions or are only just beginning to experience them.

In this context, cognitive development also plays an important role [53, 54]. Shame and guilt can only be felt if children understand that they may have evoked negative emotions in others. Therefore, it is difficult to assess whether items such as “not feeling remorseful when doing something wrong; item 18” or “feeling bad or guilty when doing something wrong; item 5” really capture CU traits in younger samples. By adolescence, children are able to anticipate feelings such as guilt and shame [55]. At this age, it becomes possible to distinguish whether a behavior is exhibited or not because a child is developmentally unable to do so or because CU traits are actually present.

Similar difficulties are evident with ICU items intended to capture not showing emotions, such as “not showing emotions to others; item 6.” In middle childhood, children increasingly prefer mental strategies for emotion regulation, such as distancing, to regulate their anger [56]. This may possibly be an alternative explanation for CU traits with high values on corresponding items. Younger children may still be in the process of developing emotional competence in general and therefore may show deficits in responding to the items.

This study points out possible misinterpretation of CU traits in younger children if developmental factors are not taken into account. The incorrect classification of normative developmental stages of children as pathological can lead to inappropriate interventions. The findings underscore the importance of considering age-related differences in emotional and cognitive development to avoid unintentional pathologization of typical behaviors. Early intervention strategies should therefore acknowledge the dynamic nature of CU traits during early childhood. By incorporating insights from developmental psychology, assessments can better account for age-specific variations in emotional and cognitive development. Before drawing conclusions on children’s CU traits, the emotional and cognitive precursor abilities of children should be examined.

Therefore, our study highlights the need for a differentiated instrument to capture CU traits that is able to distinguish CU behaviors from deviating developmental steps, especially in young samples.

### Limitations and further research

In addition to our new findings, our study’s limitations also need to be mentioned. For the present study, it should be noted that the wording of the items of the ICU differed quite slightly for the preschool teacher-reports and self-reports for children in middle childhood and adolescents. This is accompanied by a possible deviation between the raters. Whereas in middle childhood and adolescence, the children themselves were the raters, for preschool-aged children, their teachers were asked to rate the ICU. At this point, it should be noted that preschool children are not yet able to answer the ICU questions independently. While research on callous-unemotional traits in middle childhood is also incorporating self-reports [e.g., 13, 57–59], the question remains regarding children’s comprehension of the underlying concepts. However, we assume that we will arrive at more valid answers if we let the children answer the items themselves at an older age. Separately testing the factor structures for rater-based and self-report versions might introduce methodological variability, potentially diverting focus from the study’s primary aim: exploring age-related differences in interpreting CU traits. Our overarching goal is to examine measurement invariance of the ICU across the entire span of childhood and adolescence. However, as no self-report version of the ICU is available for preschool children, the only way to achieve the study objective was to use a combination of rater based and self-report assessments. Additonally, Wang et al. [60] identified cross-informants (self-report, parent-report, and teacher-report) invariance. Moreover, younger children may possess limited introspective capacities, often relying more on external observations by parents or teachers. In contrast, adolescents typically demonstrate a more nuanced understanding of their own internal emotional experiences. However, to ensure a more precise examination of the measurement invariance of the ICU, it is pertinent to utilize consistent rater sources. Futher studies could use the option of using teacher and/or parent reports for all age groups.

The field of research on the ICU instrument is ambiguous. Existing studies were able to confirm different factor structures of the ICU for varying samples [5, 13, 16]. For our study’s approach, we have chosen the three-factor solution because it is suggested by the author of the ICU [12]. In selecting the three-factor model as the primary focus, the study aims to enhance the overall comparability of findings across different age groups [e.g., 2, 15]. There are a variety of alternative models [e.g. a two-factor model, including a callousness and an uncaring factor following the procedure of Hawes et al. [5] or a two-factor model with the factors callous-unemotional and empathic-prosocial derived from Willoughby et al. [17]. However, testing alternative models for each age group could introduce complexity and hinder the ability to draw meaningful cross-age comparisons. By adhering to the established structure, the research strives for a unified framework that facilitates a more comprehensive understanding of callous-unemotional traits across the developmental spectrum. In further research, however, alternative ICU models could certainly also be tested for measurement invariance.

In addition, we used error terms correlations. However, if items have similar meanings or refer to similar concepts, this can lead to an increased likelihood of correlations between the residuals. In such cases, modeling correlated errors can help to better reflect the factor structure and reduce potential biases [61].

## Conclusion

In conclusion, our study assessed the ICU across different age groups (preschool, middle childhood, early, and late adolescence) and identified consistent structural patterns but varying interpretations of individual items. This highlights the need for a more differentiated assessment, as items could be interpreted differently across the age groups. The study challenges the assumption of uniform developmental prerequisites for understanding ICU items. Our findings underscore the difficulty in distinguishing callous-unemotional traits from normal developmental stages, especially in younger children. Nevertheless, future research is needed and should explore measurement invariance for alternative ICU models to enhance understanding and measurement across diverse populations and developmental stages.

### Electronic supplementary material

Below is the link to the electronic supplementary material.

Supplementary Material 1

## Data Availability

The data that support the findings of this study are available from the corresponding author upon reasonable request.

## Abbreviations

CFI: Comparative Fit Index
CU traits: Callous-unemotional traits
FIML: Full Information Maximum Likelihood
ICU: Inventory of Callous-Unemotional Traits
MI: Measurement Invariance
RMSEA: Root Mean Square Error of Approximation
SRMR: Standardized Root Mean Squared Residual
TLI: Tucker-Lewis Index
