# Supplementary material for: Measurement invariance of the inventory of Callous‑Unemotional traits in different age groups from preschool age to late adolescence in Germany
Source: BMC Psychol. 2024 May 27;12:298. doi: 10.1186/s40359-024-01789-4 (PMC11131252; doi:10.1186/s40359-024-01789-4)
Supplement: Supplementary file 1 — Supplementary Material 1 [file 40359_2024_1789_MOESM1_ESM.docx]

**Table S 1**

*Intercorrelations of the ICU items*

| **Item** | **1** | **2** | **3** | **4** | **5** | **6** | **7** | **8** | **9** | **10** | **11** | **12** |
| --- | --- | --- | --- | --- | --- | --- | --- | --- | --- | --- | --- | --- |
| **1** | 1 |  |  |  |  |  |  |  |  |  |  |  |
| **2** | -.099*** | 1 |  |  |  |  |  |  |  |  |  |  |
| **3** | .179*** | -.024 | 1 |  |  |  |  |  |  |  |  |  |
| **4** | -.056** | .160*** | -.104*** | 1 |  |  |  |  |  |  |  |  |
| **5** | .100*** | .017 | .225*** | -.219*** | 1 |  |  |  |  |  |  |  |
| **6** | -.483*** | .269*** | -.036 | .112*** | .052* | 1 |  |  |  |  |  |  |
| **7** | -.074*** | .068** | -.202*** | .243*** | -.153*** | .114*** | 1 |  |  |  |  |  |
| **8** | .205*** | -.001 | .200*** | -.313*** | .362*** | -.064** | -.162*** | 1 |  |  |  |  |
| **9** | -.091*** | .250*** | -.146*** | .238*** | -.156*** | .219*** | .239*** | -.062** | 1 |  |  |  |
| **10** | -.080*** | .091*** | .060** | .034 | .054* | .191*** | .009 | .040 | .158*** | 1 |  |  |
| **11** | -.078*** | .090*** | -.221*** | .208*** | -.134*** | .136*** | .254*** | -.133*** | .248*** | .069** | 1 |  |
| **12** | -.231*** | .290*** | -.108*** | .287*** | -.110*** | .381*** | .197*** | -.152*** | .304*** | .137*** | .233*** | 1 |
| **13** | .112*** | -.012 | .126*** | -.082*** | .174*** | -.013 | -.053* | .176*** | -.013 | .097*** | -.005 | -.022 |
| **14** | .353*** | -.195*** | .118*** | -.059** | .034 | -.331*** | -.013 | .121*** | -.095*** | -.102*** | -.051* | -.218*** |
| **15** | .188*** | -.038 | .497*** | -.201*** | .257*** | -.034 | -.246*** | .313*** | -.110*** | .078*** | -.188*** | -.143*** |
| **16** | .181*** | -.087*** | .242*** | -.364*** | .349*** | -.081*** | -.192*** | 428*** | -.177*** | -.005 | -.169*** | -.216*** |
| **17** | .084*** | -.031 | .248*** | -.371*** | .388*** | -.008 | -.199*** | .540*** | -.155*** | .035 | -.124*** | -.166*** |
| **18** | -.099*** | .204*** | -.115*** | .252*** | -.245*** | .156*** | .185*** | -.183*** | .271*** | .085*** | .226*** | .242*** |
| **19** | .419*** | -.023 | .183*** | -.029 | .152*** | -.246*** | -.060** | .222*** | -.061** | -.053* | -.098*** | -.201*** |
| **20** | -.074*** | .131*** | -.191*** | .161*** | -.125*** | .136*** | .212*** | -.185*** | .202*** | .027 | .291*** | .151*** |
| **21** | -.094*** | .109*** | -.132*** | .369*** | -.229*** | .112*** | .215*** | -.426*** | .173*** | .043* | .237*** | .247*** |
| **22** | -.407*** | .191*** | -.018 | .117*** | .053* | .525*** | .051* | -.056** | .140*** | .132*** | .108*** | .312*** |
| **23** | .159*** | -.120*** | .397*** | -.095*** | .128*** | -.074*** | -.158*** | .174*** | -.131*** | .035 | -.102*** | -.131*** |
| **24** | .100*** | .051* | .213*** | -.489*** | .293*** | .032 | -.097*** | .403*** | .02 | .101*** | -.056** | -.073*** |
|  | **13** | **14** | **15** | **16** | **17** | **18** | **19** | **20** | **21** | **22** | **23** | **24** |
| **14** | .204*** | 1 |  |  |  |  |  |  |  |  |  |  |
| **15** | .163*** | .157*** | 1 |  |  |  |  |  |  |  |  |  |
| **16** | .249*** | .152*** | .396*** | 1 |  |  |  |  |  |  |  |  |
| **17** | .207*** | .077*** | .374*** | .564*** | 1 |  |  |  |  |  |  |  |
| **18** | -.080*** | -.060** | -.119*** | -.226*** | -.029 | 1 |  |  |  |  |  |  |
| **19** | .099*** | .264*** | .224*** | .216*** | 157*** | -.052* | 1 |  |  |  |  |  |
| **20** | -.040 | -.061** | -.224*** | -.172*** | -.165*** | .267*** | -.033 | 1 |  |  |  |  |
| **21** | -.092*** | -.030 | -.230*** | -.318*** | -.388*** | .289*** | -.104*** | .224*** | 1 |  |  |  |
| **22** | -.029 | -.276*** | -.027 | -.042* | .003 | .135*** | -.278*** | .087*** | .114*** | 1 |  |  |
| **23** | .134*** | .175*** | .521*** | .268*** | .226*** | -.114*** | .209*** | -.205*** | -.114*** | -.105*** | 1 |  |
| **24** | .196*** | .048* | .331*** | .361*** | .403*** | -.095*** | .218*** | -.055** | -.242*** | .052* | .254*** | 1 |

*Note.* Item labels in Table 3; **p* < .05; ***p* < .01; ****p* < .001.
